# Comorbid Depression and Obesity: Correlates and Synergistic Association With Noncommunicable Diseases Among Australian Men

**DOI:** 10.5888/pcd17.190420

**Published:** 2020-07-02

**Authors:** Tilahun Nigatu Haregu, John Tayu Lee, Brian Oldenburg, Gregory Armstrong

**Affiliations:** 1 Nossal Institute for Global Health, School of Population and Global Health, University of Melbourne, Melbourne, Australia

## Abstract

**Introduction:**

Obesity and depression are among the leading causes of disease worldwide. Their bidirectional relationship often results in comorbid depression and obesity, which further increases the risk of adverse health outcomes. Further evidence is needed on the correlates and synergistic association with other noncommunicable diseases. The objective of our study was to examine the correlates and synergistic association of comorbid depression and obesity with other noncommunicable diseases in a large sample of Australian men.

**Methods:**

Our cross-sectional study used data on 13,763 men aged 18 to 55 from the first wave (2013–2014) of the Australian Ten to Men study. Body mass index was calculated from self-reported weight and height. The Patient Health Questionnaire-9 was used to assess depression. We calculated the weighted prevalence of depression, obesity, and comorbid depression and obesity and examined correlates of comorbid depression and obesity by using logistic regression. We used the synergy index to measure the synergistic association of depression and obesity with other noncommunicable diseases.

**Results:**

The weighted prevalence of depression, obesity, and comorbid depression and obesity among Australian men were 12.5%, 22.2%, and 3.7%, respectively. Age, marital status, area-level socioeconomic index, educational attainment, household income, employment status, and physical activity were significantly associated with comorbid depression and obesity. Men with comorbid depression and obesity, compared with men without comorbid depression and obesity, had 7.6 times the risk of diabetes and 6.7 times the risk of hypertension.

**Conclusion:**

Co-occurrence of depression and obesity among Australian men is associated with a set of individual- and area-level correlates and a higher risk of noncommunicable diseases. The correlates identified in our study are useful in planning interventions and screening in primary care settings.

**Summary**

**What is already known on this topic?**

Depression and obesity are strongly related to each other.

**What is added by this report?**

A complex set of individual and area-level factors is associated with comorbid depression and obesity among men in Australia.

**What are the implications for public health practice?**

Evidence of correlates and synergistic association would be useful in designing integrated and focused health promotion interventions for Australian men.

## Introduction

The increasing burden of mental health disorders and obesity is a significant public health concern globally, including in Australia (1,2). An estimated 45% of Australians experience a mental health condition in their lifetime (3). In any single year, about 1 million Australians have depression and about 2 million have anxiety (4). In 2017–2018, one in 5 Australians (20.1%) had a mental or behavioral condition; approximately two-thirds (67.0%) of Australian adults were either overweight or obese, and slightly less than one-third (31.3%) were obese (3).

Depression and obesity often co-occur. A meta-analysis of 19 studies showed a bidirectional relationship between depression and obesity (5). In that study people who were depressed had a 37% increased risk of being obese, and people who were obese had an 18% increased risk of being depressed. Only 1 study from Australia, which examined the association between body mass index (BMI) and depression among young women, was included in this meta-analysis (6). A study of the prevalence of comorbid depression and obesity in Australian general practice reported a *U*-shape relationship between BMI and depression, with a higher prevalence of depression among underweight (23%) and obese (24%) adults (7).

Noncommunicable diseases (NCDs) account for 89% of premature deaths in Australia and are major contributors to socioeconomic inequality in health (8). NCDs, especially cardiovascular disease (CVD), share many common risk factors, including modifiable lifestyle factors such as unhealthy diet, physical inactivity, harmful use of alcohol, and tobacco use. Biomedical risk factors, such as high blood pressure, diabetes, dyslipidemia, and obesity, also contribute to the development and progression of CVD. About 38% of the prevalence of CVD in Australia is attributable to overweight and obesity (9).

About 38% of the total prevalence of illness in Australia is attributable to CVD risk factors, including overweight and obesity, which account for 8.4% of the total prevalence (10). Mental health disorders, such as depression and anxiety, also have a significant association with CVD (11,12). As a result of interplay among these factors, at least 25% of Australians adults with mental health problems have chronic disease comorbidities (13).

Although the relationship between depression and obesity is considered bidirectional, the strength of the association is stronger from obesity to depression than from depression to obesity (14). In addition, sex and age moderate the relationship between depression and obesity. In a study conducted in Australia, the association was stronger among women than among men and among older people (15). The prevalence of comorbid depression was 24% among obese women and 21% among obese men. On the other hand, physical ill health is thought to mediate the relationship between obesity and depression (15).

The bidirectional relationship between depression and obesity is a public health concern because each disease alone and both diseases acting together have a strong negative effect on health and quality of life (16). Comorbid depression and obesity was shown to have a larger negative effect on quality of life than the sum of the independent effects of depression and obesity (17). The management of depression can affect obesity and vice versa. Controlled studies demonstrated that treatment of depression strongly affects body weight, although these findings are heterogeneous; the effect of treatment depends on type of antidepressant used and dose and duration of use (18).

Although the association between obesity and depression has been explored, evidence gaps still exist. First, most of the available evidence is based on studies conducted in clinical settings. As a result, limited evidence exists at community and general population levels, especially among adult men. This lack of data is important, because although the prevalence of depression is lower among men than among women in Australia, the prevalence of obesity is higher among men, and the effect of the interaction of these conditions on the risk of other NCDs has not been investigated (3).

Second, the combined effect of individual- and area-level correlates of comorbid depression and obesity have not been systematically investigated in Australia by using large population-based data sets. Third, the synergistic effects of depression and obesity on the risk of other NCDs have not been examined. Although depression and obesity are known risk factors for NCDs, we know little about their possible synergistic effect. Evidence is needed to inform prevention and treatment interventions.

Therefore, understanding the socioeconomic, behavioral, and environmental correlates of comorbid depression and obesity is essential to further understanding their complex relationship. The objective of our study was to describe individual- and area-level correlates of comorbid depression and obesity and examine their synergistic association with risk of other NCDs in a large sample of Australian men.

## Methods

### Data source

We used data from the first wave of the Australian Longitudinal Study on Male Health (the Ten to Men study). This ongoing longitudinal study, which uses a mailed survey, enables understanding of how changing life stages and circumstances affect the health and well-being of men and boys. Wave 1 recruitment occurred from October 2013 through July 2014; 15,988 males aged 10 to 55 years returned completed questionnaires. Our study group consisted of the 13,763 men among these who were aged 18 to 55. Wave 1 of the Ten to Men study received ethical clearance from the University of Melbourne Human Sciences Human Ethics Sub-Committee. Participants aged 18 to 55 provided written consent. Wave 2 data were collected in 2015-2016.

A description of the cohort, methods, and sampling design in the Ten to Men study is available elsewhere (19–21). The study has a stratified, multistage, cluster sampling design and oversamples in rural and regional areas. Wave 1 included 432 Aboriginal and Torres Strait Islander people. The study questionnaire included variables on sociodemographic characteristics, geographic location, physical and emotional health, use of health care services, health behaviors, risk and protective factors, personal and family situation, life stages and life events, and social and environmental factors.

### Variables and measurement

The questionnaire for the first wave of the Ten to Men study is available online (https://tentomen.org.au/sites/default/files/adult_survey_final_with_variable_names.pdf). The key variables of interest in our study were obesity and depression. We used BMI (body weight in kilograms divided by height in meters squared [kg/m^2^]), calculated from self-reported body weight and standing height, to measure obesity. We classified men with a BMI of 30 kg/m^2^ or more as obese. For depression, the Ten to Men study used the Patient Health Questionnaire (PHQ-9), which assesses depression on the basis of 9 symptoms (22). The questionnaire scores each of the 9 symptoms on a frequency scale from 0 (not at all) to 3 (nearly every day), and the sum of the scores determines the presence and the degree of depression. We considered a PHQ-9 score of 10 or more to indicate moderate-to-severe depression. Other variables included in our analysis were sociodemographic factors (age, marital status, educational attainment, employment status, and combined annual household income), area-level factors (using the Socio-Economic Indexes for Areas [SEIFA] [23] to measure socioeconomic advantage and disadvantage), behavioral factors (current smoking, alcohol misuse [using the Alcohol Use Disorders Identification Test (24)]), physical activity (using Australia’s Physical Activity and Sedentary Behavior Guidelines for Adults [25]), number of fruit and vegetable servings per day, and the presence of NCDs other than obesity and depression: CVD (hypertension, myocardial infarction, heart failure, stroke, angina), diabetes, cancer, chronic respiratory diseases (chronic obstructive pulmonary disease, chronic bronchitis, asthma), cataracts, high cholesterol, and arthritis. The Ten to Men study assessed the presence of NCDs by using 2 questions: “Has a doctor or other health professional ever told you that you had this condition?” and “Have you been treated for or had any symptoms of this condition in the past 12 months?” We used data from the second question in our study. We measured all variables at the individual and household level, except for SEIFA and annual household income, which we measured at the area and household level, respectively.

### Data analysis

We summarized the weighted prevalence of depression, obesity, and comorbid depression and obesity by sociodemographic characteristics. We used sampling weights, computed by the Ten to Men study, according to the inverse probability of selection (26). We examined correlates of depression, obesity, and comorbid depression and obesity by using multiple logistic regression models. We tabulated adjusted odds ratios (ORs) and 95% confidence intervals (CIs) for correlates of depression, obesity, and comorbid depression and obesity. To assess the effect of SEIFA (in percentiles) on the correlates of comorbid depression and obesity, we conducted stratified analysis by SEIFA quartiles (first quartile, 1–28; second quartile, 29–51, third quartile, 52–69, fourth quartile, 70–100). In this index, the higher the score, the greater the socioeconomic advantage. We used the synergy index (17) to assess the synergistic effect of depression and obesity on the risk of other NCDs. We calculated the synergy index as the ratio of the combined effects of comorbid depression and obesity to the sum of the individual effects of depression and obesity (27). We calculated 95% CIs for the synergy index by using the delta method. This method used a standard error of the synergy index that was derived from the regression coefficients and covariance of the effects of depression, obesity, and comorbid depression and obesity on each of the NCDs. We tested for multicollinearity of all covariates; the variance inflation factors were all less than 5, indicating that the assumption of reasonable independence among predictor variables was met. We analyzed all data in Stata version 15.0 (StataCorp LLC).

## Results

About half (51.8%) of 13,763 men in our study were younger than 40 (Table 1). Almost two-thirds (65.3%) were married. One-quarter (24.9%) had less than a high school diploma, and 15.7% were unemployed at the time of the survey. One in 5 (19.2%) men were current smokers, 2 in 5 (38.4%) misused alcohol, and 2 in 3 (66.8%) had inadequate consumption of fruit and vegetables.

**Table 1:** Self-Reported Characteristics of Men Aged 18 to 55 Participating in Wave 1 of the Australian Longitudinal Study on Male Health (Ten to Men Study) (N = 13,763), October 2013–July 2014^a^

Characteristic/Factor	Depression Only	Obesity Only	Comorbid Depression and Obesity	Total (95% CI)
**No. (% of study population)**	982 (8.2)	2,461 (18.5)	510 (3.7)	—
**Sociodemographic Characteristics**				
**Age, y**				
18–29	11.0 (9.6–12.5)	11.1 (9.7–12.6)	2.5 (1.9–3.3)	25.1 (24.1–26.1)
30–39	8.3 (6.9–9.8)	16.0 (14.5–17.6)	3.8 (3.0–4.7)	26.7 (25.7–27.7)
40–49	6.4 (5.5–7.5)	23.5 (21.7–25.3)	4.1 (3.4–4.9)	30.6 (29.5–31.7)
50–55	7.8 (6.3–9.6)	23.1 (20.9–25.6)	4.7 (3.7–5.8)	17.6 (16.8–18.5)
**Marital status**				
Never married	12.5 (11.0–14.2)	13.2 (11.7–14.9)	4.6 (3.7–5.6)	28.0 (27.0–29.1)
Divorced/widowed/separated	14.3 (11.5–17.7)	20.7 (17.3–24.7)	7.1 (5.3–9.6)	6.6 (6.1–7.2)
Currently married	6.0 (5.3–6.7)	20.4 (19.3–21.5)	3.1 (2.6–3.5)	65.3 (64.2–66.5)
**Educational attainment**				
≤High school	11.5 (10.0–13.3)	20.2 (18.3–22.2)	5.9 (4.9–7.1)	24.9 (23.9–25.9)
Diploma or certificate	8.4 (7.4–9.5)	21.6 (20.2–23.1)	4.0 (3.4–4.8)	43.2 (42.0–44.4)
Bachelor’s degree or above	5.5 (4.5–6.7)	12.8 (11.5–14.4)	1.8 (1.4–2.5)	30.1 (29.0–31.2)
Other	11.5 (7.0–18.2)	19.6 (11.9–30.5)	5.5 (3.0–9.7)	1.9 (1.6–2.2)
**Combined annual household income, A$**				
<40,000	21.1 (17.5–25.2)	17.2 (14.3–20.5)	7.9 (6.1–10.1)	11.4 (10.6–12.2)
40,000–79,999	9.1 (7.8–10.6)	18.9 (17.1–20.9)	5.4 (4.5–6.6)	26.7 (25.6–27.8)
≥80,000	5.2 (4.6–6.0)	19.2 (18.0–20.4)	2.5 (2.1–3.0)	61.9 (60.7–63.1)
**Employment status**				
Employed	6.4 (5.8–7.0)	18.7 (17.8–19.7)	2.8 (2.5–3.3)	84.3 (83.4–85.2)
Unemployed	19.3 (16.5–22.4)	16.9 (14.5–19.6)	9.3 (7.7–11.1)	15.7 (14.8–16.6)
**Lifestyle and Behavioral Factors**				
**Current smoking**				
No	6.5 (5.9–7.2)	18.7 (17.7–19.7)	3.3 (2.9–3.8)	80.8 (79.9–81.7)
Yes	16.0 (13.9–18.3)	17.3 (15.4–19.4)	5.6 (4.6–6.8)	19.2 (18.3–20.1)
**Alcohol misuse**				
No	6.9 (6.1–7.9)	19.3 (18.1–20.6)	3.3 (2.8–3.8)	61.6 (60.4–62.7)
Yes	9.6 (8.6–10.7)	18.0 (16.5–19.6)	4.2 (3.5–5.0)	38.4 (37.3–39.6)
**Fruit and vegetable intake, servings per day**				
Adequate (≥5)	9.0 (8.2–10.0)	18.8 (17.8–20.0)	4.0 (3.6–4.6)	33.2 (32.2–34.3)
Inadequate (<5)	6.7 (5.8–7.8)	17.7 (16.2–19.3)	3.1 (2.5–3.9)	66.8 (65.7–67.8)
**Physical activity, per week**				
Sedentary (0 min and 0 sessions)	10.2 (8.3–12.4)	25.7 (22.9–28.7)	8.2 (6.6–10.3)	13.6 (12.8–14.4)
Insufficiently active (<150 min or <5 sessions)	9.3 (7.9–10.9)	20.0 (18.2–21.8)	4.9 (4.1–5.9)	29.1 (28.0–30.2)
Sufficiently active (>150 min in >5 sessions)	7.0 (6.3–7.9)	16.7 (15.5–18.0)	2.4 (2.0–2.9)	57.4 (56.2–58.6)
**Chronic conditions^b^**				
Depression (PHQ-9 ≥10)^c^	—	31.2 (28.2–34.2)	—	12.5 (11.8–13.3)
Obesity (BMI ≥30 kg/m^2^)^d^	16.8 (15.2–18.6)	—	—	22.2 (21.3–23.2)
Cardiovascular disease or stroke	2.7 (1.8–4.1)	2.4 (1.7–3.4)	5.6 (3.4–9.0)	1.7 (1.4–2.1)
Diabetes	3.8 (2.7–5.2)	6.6 (5.4–8.1)	14.4 (10.9–18.7)	3.2 (2.8–3.6)
Hypertension	11.7 (9.3–14.5)	20.5 (18.4–22.7)	32.7 (27.7–38.2)	9.9 (9.3–10.6)
Asthma	14.8 (12.2–18.0)	9.6 (8.2–11.1)	20.3 (15.9–25.6)	9.1 (8.5–9.8)
Arthritis	10.9 (8.5–13.9)	9.5 (8.0–11.3)	19.4 (15.2–24.4)	6.9 (6.4–7.6)
High cholesterol	9.4 (7.6–11.6)	13.8 (12.0–15.8)	26.1 (21.3–31.5)	8.8 (8.1–9.5)

a Values are weighted % (95% CI) unless otherwise noted.

b Survey participants answered the following yes–no question: “Have you been treated for or had any symptoms of this condition in the past 12 months?”

c The study used the Patient Health Questionnaire (PHQ-9) to assess depression on the basis of 9 symptoms (22). The questionnaire scores each of the 9 symptoms on a frequency scale from 0 (not at all) to 3 (nearly every day), and the sum of the scores determines the presence and the degree of depression. We considered a PHQ-9 score of 10 or more to indicate moderate-to-severe depression.

d We used BMI, calculated from self-reported body weight and standing height (body weight in kg divided by height in meters squared [kg/m^2^]), to measure obesity.

One in 8 (12.5%) men had depression. About 1 in 5 (22.2%) were obese. Nearly one-third (31.2%) of men with depression were obese, and 16.8% of men who were obese had depression. The weighted prevalence of comorbid depression and obesity was 3.7%.

### Correlates of comorbid depression and obesity

The risk of depression decreased with age, whereas the risk of obesity increased with age. Higher educational attainment was associated with a lower risk of depression. Unemployment was associated with higher risk of depression. The prevalence of comorbid depression and obesity was significantly higher among men aged 30 to 39, 40 to 49, and 50 to 55 than among men aged 18 to 29. Being single or never married, being in the first SEIFA quartile, and being sedentary were significantly associated with higher risk of comorbid depression and obesity (Table 2).

**Table 2:** Prevalence and Correlates of Depression, Obesity, and Comorbid Depression and Obesity Among Men Aged 18 to 55 Participating in Wave 1 of the Australian Longitudinal Study on Male Health (Ten to Men Study) (N = 13,763), October 2013–July 2014^a^

Characteristic/Factor	Depression Only	Obesity Only	Comorbid Depression and Obesity
**Sociodemographic Characteristics**			
**Age, y**			
18–29	1.00 [Reference]	1.00 [Reference]	1.00 [Reference]
30–39	0.86 (0.64–1.16) [.32]	1.47 (1.14–1.91) [.004]	4.46 (2.60–7.66) [<.001]
40–49	0.68 (0.50–0.91) [.01]	2.34 (1.82–3.01) [<.001]	4.58 (2.59–8.09) [<.001]
50–55	0.61 (0.43–0.86) [.005]	2.22 (1.7–2.91) [<.001]	4.53 (2.50–8.21) [<.001]
**Marital status**			
Never married	1.00 [Reference]	1.00 [Reference]	1.00 [Reference]
Divorced/widowed/separated	1.36 (0.93–1.98) [.11]	0.90 (0.63–1.30) [.59]	0.71 (0.40–1.25) [.23]
Currently married	0.78 (0.60–1.02) [.07]	1.04 (0.82–1.31) [.78]	0.48 (0.31–0.75) [.001]
**Educational attainment**			
≤High school	1.00 [Reference]	1.00 [Reference]	1.00 [Reference]
Diploma or certificate	0.92 (0.72–1.16) [.48]	0.91 (0.76–1.10) [.35]	0.72 (0.51–1.01) [.06]
Bachelor’s degree or above	0.75 (0.55–1.05) [.09]	0.60 (0.48–0.75) [<.001]	0.42 (0.27–0.67) [<.001]
Other	1.18 (0.71–1.98) [.52]	0.96 (0.42–2.18) [.92]	1.06 (0.46–2.45) [.89]
**Combined annual household income, A$**			
<40,000	1.00 [Reference]	1.00 [Reference]	1.00 [Reference]
40,000–79,999	0.69 (0.50–0.94) [.02]	1.15 (0.82–1.61) [.42]	0.82 (0.49–1.38) [.46]
≥80,000	0.48 (0.34–0.66) [<.001]	1.22 (0.88–1.68) [.24]	0.54 (0.32–0.93) [.03]
**Employment status**			
Employed	1.00 [Reference]	1.00 [Reference]	1.00 [Reference]
Unemployed	2.87 (2.17–3.78) [<.001]	1.09 (0.82–1.45) [.56]	3.33 (2.25–4.93) [<.001]
**SEIFA quartiles^b^**			
First	1.00 [Reference]	1.00 [Reference]	1.00 [Reference]
Second	1.03 (0.78–1.35) [.85]	0.81 (0.66–1.00) [.048]	0.95 (0.64–1.40) [.80]
Third	0.84 (0.62–1.12) [.23]	0.75 (0.61–0.93) [.007]	0.85 (0.58–1.27) [.43]
Fourth	0.96 (0.72–1.27) [.76]	0.54 (0.44–0.68) [<.001]	0.36 (0.23–0.56) [<.001]
**Lifestyle and Behavioral Factors**			
**Current smoking**			
No	1.00 [Reference]	1.00 [Reference]	1.00 [Reference]
Yes	1.68 (1.33–2.12) [<.001]	0.85 (0.69–1.04) [.12]	0.81 (0.55–1.17) [.26]
**Alcohol misuse**			
No	1.00 [Reference]	1.00 [Reference]	1.00 [Reference]
Yes	1.43 (1.16–1.76) [.001]	0.94 (0.80–1.10) [.44]	1.26 (0.92–1.73) [.15]
**Fruit and vegetable intake, servings per day**			
Adequate (≥5)	1.00 [Reference]	1.00 [Reference]	1.00 [Reference]
Inadequate (<5)	1.13 (0.90–1.42) [.30]	1.04 (0.89–1.22) [.59]	1.10 (0.79–1.53) [.57]
**Physical activity, per week**			
Sedentary (0 min and 0 sessions)	1.00 [Reference]	1.00 [Reference]	1.00 [Reference]
Insufficiently active (<150 min or <5 sessions)	0.87 (0.65–1.16) [.34]	0.78 (0.62–0.98) [.04]	0.59 (0.40–0.87) [.008]
Sufficiently active (>150 min in >5 sessions)	0.63 (0.47–0.84) [.001]	0.68 (0.55–0.84) [<.001]	0.27 (0.17–0.41) [<.001]

a All values are odds ratio (95% confidence interval) [*P* value]. All odds ratios were adjusted for other characteristics included in this table. Except for SEIFA quartiles, all data were self-reported.

b We used the Socio-Economic Indexes for Areas (SEIFA) to measure socioeconomic status (23). Data were stratified by SEIFA quartiles (first quartile, 1–28; second quartile, 29–51, third quartile, 52–69, fourth quartile, 70–100). In this index, the higher the score (and quartile), the greater the socioeconomic advantage.

The stratified analyses across SEIFA quartiles showed that age, employment status, and physical activity were consistently and significantly associated with comorbid depression and obesity across all quartiles. Married men had reduced risk of comorbid depression and obesity compared with never-married men in lower SEIFA quartiles. Higher educational attainment was significantly associated with reduced risk of comorbidity in the first and third SEIFA quartiles. Similarly, higher income was also associated with reduced risk of comorbidity in the third and fourth SEIFA quartiles (Table 3).

**Table 3:** Correlates of Comorbid Depression and Obesity, by SEIFA Quartiles,^a^ Among Men Aged 18 to 55 Participating in Wave 1 of the Australian Longitudinal Study on Male Health (Ten to Men Study) (N = 13,763), October 2013–July 2014^b^

Characteristic/Factor	1st Quartile	2nd Quartile	3rd Quartile	4th Quartile
**Age, y**				
18–29	1.00 [Reference]	1.00 [Reference]	1.00 [Reference]	1.00 [Reference]
30–39	4.32 (1.84–10.16) [.001]	3.54 (1.12–11.21) [.03]	5.30 (1.62–17.32) [.006]	5.69 (1.20–27.02) [.03]
40–49	2.61 (1.06–6.40) [.04]	6.36 (1.98–20.39) [.002]	5.69 (1.57–20.60) [.008]	7.69 (1.58–37.29) [.01]
50–55	4.33 (1.74–10.76) [.002]	5.67 (1.73–18.59) [.004]	3.56 (0.94–13.50) [.06]	5.28 (0.98–28.56) [.05]
**Marital status**				
Never married	1.00 [Reference]	1.00 [Reference]	1.00 [Reference]	1.00 [Reference]
Previously married	0.67 (0.30–1.52) [.34]	0.51 (0.16–1.61) [.25]	0.89 (0.26–3.06) [.86]	1.81 (0.39–8.36) [.44]
Currently married	0.51 (0.27–0.97 [.04]	0.40 (0.17–0.96) [.04]	0.51 (0.18–1.46) [.21]	0.42 (0.13–1.31) [.13]
**Educational attainment**				
≤High school	1.00 [Reference]	1.00 [Reference]	1.00 [Reference]	1.00 [Reference]
Diploma or certificate	0.75 (0.42–1.33) [.32]	0.97 (0.53–1.80) [.93]	0.61 (0.31–1.19) [.15]	0.45 (0.15–1.30) [.14]
Bachelor’s degree or above	0.32 (0.12–0.84) [.02]	0.73 (0.33–1.61) [.43]	0.31 (0.13–0.77) [.01]	0.41(0.14–1.16) [.09]
Other	0.77 (0.13–4.48) [.77]	1.05 (0.25–4.49) [.94]	4.08 (1.01–16.41) [.048]	—c
**Combined annual household income, A$**				
<40,000	1.00 [Reference]	1.00 [Reference]	1.00 [Reference]	1.00 [Reference]
40,000–79,999	1.04 (0.48–2.23) [.92]	1.25 (0.49–3.15) [.64]	0.51 (0.17–1.54) [.23]	0.20 (0.05–0.91) [.04]
≥80,000	0.55 (0.23–1.30) [.17]	0.80 (0.32–1.97) [.62]	0.32 (0.10–1.01) [.05]	0.59 (0.18–1.95) [.39]
**Employment status**				
Employed	1.00 [Reference]	1.00 [Reference]	1.00 [Reference]	1.00 [Reference]
Unemployed	3.06 (1.64–5.71) [<.001]	3.70 (1.76–7.78) [.001]	3.42 (1.47–7.92) [.004]	4.71 (1.68–13.21) [.003]
**Current smoking**				
No	1.00 [Reference]	1.00 [Reference]	1.00 [Reference]	1.00 [Reference]
Yes	0.89 (0.53–1.50) [.66]	0.70 (0.36–1.38) [.30]	1.06 (0.43–2.60) [.90]	0.58 (0.20–1.69) [.32]
**Alcohol misuse**				
No	1.00 [Reference]	1.00 [Reference]	1.00 [Reference]	1.00 [Reference]
Yes	1.05 (0.61–1.81) [.86]	1.07 (0.56–2.05) [.84]	1.58 (0.80–3.09) [.18]	1.73 (0.82–3.63) [.15]
**Fruit and vegetable intake, servings per day**				
≥5	1.00 [Reference]	1.00 [Reference]	1.00 [Reference]	1.00 [Reference]
<5	0.79 (0.45–1.40) [.43]	1.20 (0.60–2.41) [.60]	1.67 (0.87–3.17) [.12]	1.47 (0.68–3.16) [.32]
**Physical activity**				
Sedentary (0 min and 0 sessions)	1.00 [Reference]	1.00 [Reference]	1.00 [Reference]	1.00 [Reference]
Insufficiently active (<150 min or <5 sessions)	0.62 (0.32–1.23) [.17]	0.36 (0.18–0.73) [.004]	0.58 (0.28–1.20) [.14]	0.84 (0.30–2.35) [.74]
Sufficiently active (>150 min in >5 sessions)	0.26 (0.12–0.56) [.001]	0.28 (0.14–0.60) [.001]	0.27 (0.12–0.58) [.001]	0.17 (0.05–0.55) [.003]

a We used the Socio-Economic Indexes for Areas (SEIFA) to measure socioeconomic status (23). Data were stratified by SEIFA quartiles (first quartile, 1–28; second quartile, 29–51, third quartile, 52–69, fourth quartile, 70–100). In this index, the higher the score (and quartile), the greater the socioeconomic advantage.

b All values are odds ratio (95% confidence interval) [*P* value].

c Numbers too small to make calculation.

### Synergistic association of depression and obesity with other NCDs

We found a strong positive association between comorbid depression and obesity and the risk of other NCDs. Men with comorbid depression and obesity had 7.6 times the risk of diabetes, 6.7 times the risk of hypertension, and 4.3 times the risk of high cholesterol, compared with men without comorbid depression and obesity. The analysis of synergistic effects showed a 68% excess risk of diabetes, 57% excess risk of hypertension, and more than twice the excess risk of arthritis and high cholesterol among men with comorbid depression and obesity, compared with the sum of the independent risks from depression and obesity (Table 4).

**Table 4:** Association Between Comorbid Depression and Obesity and Other Noncommunicable Diseases Among Men Aged 18 to 55 Participating in Wave 1 of the Australian Longitudinal Study on Male Health (Ten to Men Study) (N = 13,763), October 2013–July 2014^a^

Noncommunicable Disease	Odds Ratio (95% Confidence Interval) [*P* Value]	Synergy Index (95% Confidence Interval)^b^
**Cardiovascular disease/stroke**		
Depressive symptoms only	1.54 (0.75–3.16) [.24]	—
Obesity only	1.25 (0.71–2.20) [.45]	—
Depressive symptoms and obesity	1.86 (0.70–5.00) [.22]	1.10 (0.11–11.31)
**Diabetes**		
Depressive symptoms only	2.26 (1.19–4.28) [.01]	—
Obesity only	3.67 (2.44–5.53) [<.001]	—
Depressive symptoms and obesity	7.62 (4.51–12.87) [<.001]	1.68 (0.92–3.08)
**Hypertension**		
Depressive symptoms only	2.29 (1.61–3.26) [<.001]	—
Obesity only	3.36 (2.69–4.19) [<.001]	—
Depressive symptoms and obesity	6.74 (4.73–9.60) [<.001]	1.57 (1.02–2.44)
**Asthma**		
Depressive symptoms only	1.81 (1.29–2.52) [.001]	—
Obesity only	1.36 (1.08–1.72) [.01]	—
Depressive symptoms and obesity	2.69 (1.81–4.00) [<.001]	1.45 (0.66–3.19)
**Arthritis**		
Depressive symptoms only	1.42 (0.94–2.13) [.09]	—
Obesity only	1.35 (1.03–1.77) [.03]	—
Depressive symptoms and obesity	3.02 (2.07–4.40) [<.001]	2.62 (0.98–7.01)
**High cholesterol**		
Depressive symptoms only	1.72 (1.18–2.51) [.004]	—
Obesity only	1.90 (1.48–2.43) [<.001]	—
Depressive symptoms and obesity	4.31 (2.93–6.34) [<.001]	2.04 (1.11–3.75)

a Outcome variables were each of the noncommunicable diseases. Main predictor was combination of depression and obesity. All models were adjusted for the Socio-Economic Indexes for Areas (23), age, income, marital status, educational attainment, smoking, alcohol, physical activity, and employment.

b The synergy index shows the excess risk from comorbid depression and obesity when compared with the sum of independent risks from depression and obesity. For example, a synergy index of 2 means the risk of high cholesterol among men with comorbid depression and obesity is 2 times the sum of independent risks from depression and obesity.

The predicted prevalence of NCDs was higher among men with comorbid depression and obesity than among men that had neither condition, men who had depression only, and men who had obesity only (Figure). The prevalence of hypertension, high cholesterol, asthma, and arthritis was higher than the prevalence of other chronic conditions included in our analysis. Stratification of these effects by SEIFA quartiles showed that the effects were higher in low SEIFA quartiles, especially for stroke, hypertension, arthritis, and high cholesterol.

**Figure:**
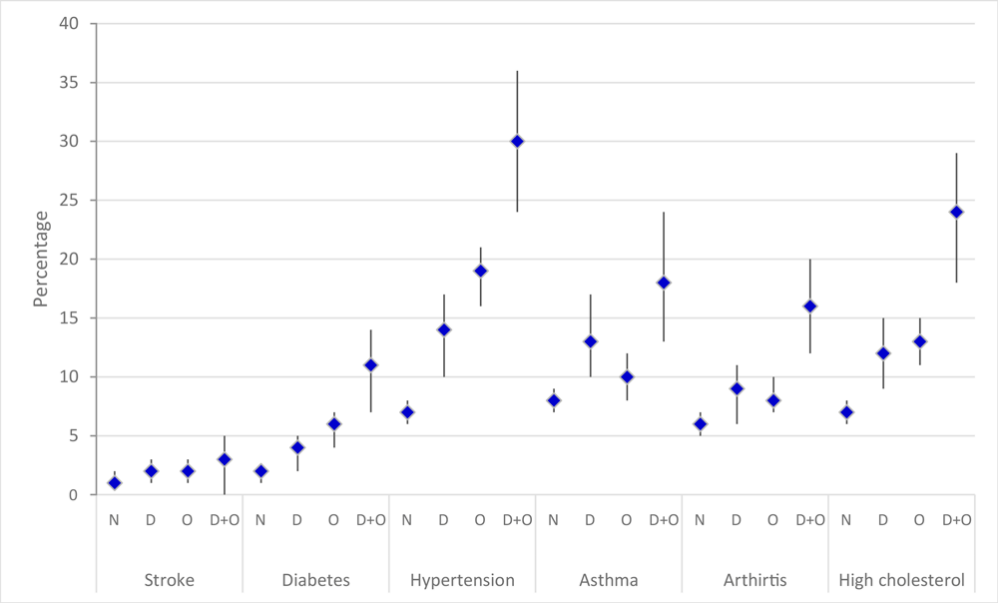
Predicted prevalence of noncommunicable diseases by neither condition (N), depression only (D), obesity only (O), and depression and obesity (D + O) among men aged 18–55 participating in wave 1 of the Ten to Men study on male health in Australia, 2013–2014. Error bars indicate 95% confidence intervals.

**Table:** 

Condition	Neither Depression nor Obesity	Depression Only	Obesity Only	Diabetes and Obesity
**Stroke**	1.5 (1.0–2.0)	2.2 (1.0–3.5)	1.8 (1.1–2.6)	2.7 (0.5–4.8)
**Diabetes**	1.6 (1.2–2.1)	3.6 (1.7–5.4)	5.6 (4.2–7.0)	10.6 (6.8–14.4)
**Hypertension**	6.9 (5.9–7.8)	13.8 (10.4–17.3)	18.6 (16.4–20.8)	29.8 (23.9–35.7)
**Asthma**	8.6 (6.8–7.7)	13.1 (9.6–16.6)	10.2 (8.5–12.0)	18.4 (12.8–23.9)
**Arthritis**	6.4 (5.4–7.3)	8.6 (5.8–11.5)	8.3 (6.7–9.9)	15.9 (11.7–20.1)
**High cholesterol**	7.4 (6.4–8.4)	11.8 (8.5–15.2)	12.8 (10.8–14.8)	23.6 (18.0–29.2)

## Discussion

We found a 3.7% overall prevalence of comorbid depression and obesity among Australian men aged 18 to 55 years. However, we observed a higher prevalence (31.2%) of obesity among men with depression than among men in the entire study population (18.5%). We also found a set of factors associated with comorbid depression and obesity. Age, employment status, and physical activity were consistently associated with comorbid depression and obesity across all levels of socioeconomic status categories. Moreover, we demonstrated a significant association between comorbid depression and obesity and excess risk of other NCDs.

A systematic review and meta-analysis of 9 studies reported that people with obesity were 32% (36% among women and 8% among men) more likely than people with normal BMI to have depression (28). Our study found a 62% increased risk of depression among men who were obese. The difference in findings between the systematic review and our study could be due to several factors. The 9 studies in the systematic review were from the United States, Canada, and Norway. They included both men and women and used different scales to assess depression. Our study was limited to Australian men and used the PHQ-9 to assess depression. One study in the systematic review used the PHQ-9 to assess depression in the National Health and Nutrition Examination Survey and estimated a 2.5 times higher risk among adults with obesity (29), higher than our estimate of risk. The difference between estimates could be due to differences in population characteristics.

The prevalence of depression among obese patients was reported to be 23% in a study of general practice clinics in Australia (7). Our study found a 17% prevalence of depression among obese men. Although the study populations differed, both studies used the PHQ-9 to assess depression, and the prevalence in the study of obese patients in general practice is not too far off the prevalence found in our study. In Mexico, a study that used the Hamilton Depression Rating Scale to assess depression in patients with type 2 diabetes reported a nearly 50% prevalence among obese patients (30). This higher prevalence could be due to the effect of type 2 diabetes on depression.

Our study found that older age and unemployment were associated with a higher risk of comorbid depression and obesity. We also showed a decline in the risk of depression but an increase in the risk of obesity as age increased. This finding is consistent with the findings reported by other studies (31,32). The higher risk of comorbid depression and obesity among unemployed men than among employed men in this study could have been due to the stronger effect of unemployment on the risk of depression among men with obesity than among men without obesity.

On the other hand, our study showed that higher educational attainment was associated with lower risk of obesity and comorbid depression and obesity. However, some studies indicated that high educational attainment was associated with greater risk of comorbid depression and obesity, with prominent effects among women (33). This difference could be related to the stronger relationship between depression and obesity among women than among men. Higher household income was negatively associated with depression and comorbid depression and obesity. Similar studies reported higher levels of mental health disorders among people with low educational attainment and low household income (34).

In our study, physical activity was associated with a lower risk of depression, obesity, and comorbid depression and obesity. This finding is consistent with findings from other studies that show the negative effects of depression on physical activity, which in turn increases the risk of obesity (35,36). However, we did not find any significant associations between smoking, alcohol use, or consumption of fruit and vegetables and comorbid depression and obesity.

In addition to the individual-level correlates, the area-level factor, SEIFA, was significantly associated with the risk of obesity and comorbid depression and obesity. Men in the lower SEIFA quartiles had a higher prevalence of depression, obesity, and comorbid depression and obesity. Similar studies in Australia reported a higher risk of obesity among socially disadvantaged people (37). The effect of SEIFA on risk of comorbid depression and obesity needs to be further explored, because other factors may explain this association.

In this study, we found a significant association between comorbid depression and obesity and excess risk of other NCDs, such as diabetes, hypertension, and high cholesterol. This association was moderated by socioeconomic status. The excess risk of other NCDs among men with comorbid depression and obesity has implications for public health: prevention, early detection, and management of NCDs are needed for men with this comorbidity. Evidence of correlates and synergistic association would be useful in designing integrated and focused health promotion interventions However, further research, preferably longitudinal research, is needed to investigate the synergistic effect of depression and obesity on the risk of other NCDs.

Our study had several limitations. First, because the study design was cross-sectional, we could not establish a sequence of events for the onset of depression, obesity, and other NCDs. Establishing this sequence would affect the direction of the association between depression and obesity. Second, the Ten to Men study was not designed to study comorbid depression and obesity or its effects on the risk of other NCDs. Consequently, the number of men with other NCDs was small, and this small number affected the power of the study, especially for determining the significance of the synergy index. Third, self-reported weight and height were used to calculate BMI, which is less accurate than height and weight measured by health care professionals. Similarly, depression was assessed by using the PHQ-9, which is based on self-report. Although this scale is well validated, the possibility of social desirability bias cannot be ruled out. Finally, this study focused on men, and the findings cannot be generalized to the overall population or women.

Our study found that the overall prevalence of comorbid depression and obesity among Australian men was 3.7%. Comorbid depression and obesity among Australian men was associated with a set of individual-level sociodemographic factors, including age, marital status, educational attainment, household income, and employment status. Among the behavioral factors studied, physical activity was significantly associated with comorbid depression and obesity. We also found an inverse association between SEIFA, an area-level factor, and comorbid depression and obesity. Comorbid depression and obesity was associated with excess risk of other NCDs. Moreover, we showed that comorbid depression and obesity was associated with a risk of NCDs that was higher than the risk found by summing the independent effects of depression and obesity. The correlates identified in our study are useful in planning interventions and screening in primary care settings. Further research is needed to explain the mechanisms that underpin these relationships.
